# Comprehensive Network Analysis of Anther-Expressed Genes in Rice by the Combination of 33 Laser Microdissection and 143 Spatiotemporal Microarrays

**DOI:** 10.1371/journal.pone.0026162

**Published:** 2011-10-26

**Authors:** Koichiro Aya, Go Suzuki, Keita Suwabe, Tokunori Hobo, Hirokazu Takahashi, Katsuhiro Shiono, Kentaro Yano, Nobuhiro Tsutsumi, Mikio Nakazono, Yoshiaki Nagamura, Makoto Matsuoka, Masao Watanabe

**Affiliations:** 1 Bioscience and Biotechnology Center, Nagoya University, Nagoya, Japan; 2 Division of Natural Science, Osaka Kyoiku University, Kashiwara, Japan; 3 Graduate School of Life Sciences, Tohoku University, Sendai, Japan; 4 Graduate School of Bioresources, Mie University, Tsu, Japan; 5 Graduate School of Agricultural and Life Sciences, The University of Tokyo, Tokyo, Japan; 6 School of Agriculture, Meiji University, Kawasaki, Japan; 7 Graduate School of Bioagricultural Sciences, Nagoya University, Nagoya, Japan; 8 National Institute of Agrobiological Sciences, Tsukuba, Japan; 9 Faculty of Science, Tohoku University, Sendai, Japan; Iwate University, Japan

## Abstract

Co-expression networks systematically constructed from large-scale transcriptome data reflect the interactions and functions of genes with similar expression patterns and are a powerful tool for the comprehensive understanding of biological events and mining of novel genes. In *Arabidopsis* (a model dicot plant), high-resolution co-expression networks have been constructed from very large microarray datasets and these are publicly available as online information resources. However, the available transcriptome data of rice (a model monocot plant) have been limited so far, making it difficult for rice researchers to achieve reliable co-expression analysis. In this study, we performed co-expression network analysis by using combined 44 K agilent microarray datasets of rice, which consisted of 33 laser microdissection (LM)-microarray datasets of anthers, and 143 spatiotemporal transcriptome datasets deposited in RicexPro. The entire data of the rice co-expression network, which was generated from the 176 microarray datasets by the Pearson correlation coefficient (PCC) method with the mutual rank (MR)-based cut-off, contained 24,258 genes and 60,441 genes pairs. Using these datasets, we constructed high-resolution co-expression subnetworks of two specific biological events in the anther, “meiosis” and “pollen wall synthesis”. The meiosis network contained many known or putative meiotic genes, including genes related to meiosis initiation and recombination. In the pollen wall synthesis network, several candidate genes involved in the sporopollenin biosynthesis pathway were efficiently identified. Hence, these two subnetworks are important demonstrations of the efficiency of co-expression network analysis in rice. Our co-expression analysis included the separated transcriptomes of pollen and tapetum cells in the anther, which are able to provide precise information on transcriptional regulation during male gametophyte development in rice. The co-expression network data presented here is a useful resource for rice researchers to elucidate important and complex biological events.

## Introduction

Recent developments in high-throughput microarray, next-generation sequencing, proteomic analysis, and the accumulated functional genomics data across species have enabled us to utilize integrated large-scale data of gene-expression, protein-protein interaction, and phenotype. There is now an increasing need for integrated analysis at a system biology level, to gain an understanding of the complex relationships between gene-product interactions and biological events (e.g. phenotype). An *in-silico*-derived co-expression network is constructed from large-scale gene expression profiles, and is based on the assumption that genes with similar expression patterns are likely to interact with each other at the molecular or physiological level. In some model plants as well as animals, this strategy has been broadly used to predict integrated networks in association with protein-protein interaction data [1], the structural information on metabolic pathways [2]–[4], and the functions of gene products [5].

To date, a model plant, *Arabidopsis thaliana*, has been subjected to thousands of microarray experiments and the results have been deposited in publicly-available online databases [6]. By using these very large expression datasets, high-resolution co-expression networks have been constructed and these are available as online information resources in *Arabidopsis*. Such useful information resources have enabled *Arabidopsis* researchers to identify novel factors involved in cell wall synthesis [3], [4] and the aliphatic glucosinolate biosynthesis pathway [5]. On the other hand, in a model crop for the grass family, rice (*Oryza sativa*), there are few resources for co-expression network analysis, because of inadequate proliferation of available rice microarray datasets derived from various different platforms and a low number of datasets with few examples of publicly available data [7]. Moreover, in rice, there have been fewer case studies to estimate the effectiveness of co-expression networks, compared to those in *Arabidopsis*. Thus, a reliable large-scale study of co-expression network analysis is required to drive forward rice research at this time.

Male gametophyte development might be a good target for co-expression network analysis in rice, because pollen transcriptome can be easily and precisely analyzed using larger rice flowers than those in *Arabidopsis.* In flowering plants, pollen development takes place within a male reproductive organ, the anther, and is controlled precisely by four sporophytic cell layers of the anther (tapetum, middle layer, endothecium and epidermis), which surround the gametophytic pollen grains [8]. After differentiation of the male germline, pollen mother cells form tetrads of haploid microspores via meiosis, and the tetrad microspores are connected to each other by a callose wall. Tapetum cells play an important role in degradation of the callose wall, which allows the microspores to be released into the anther locule. The released microspores subsequently mature into pollen grains through cell division and pollen wall formation. During the course of pollen maturation, the tapetum starts to degenerate and provides the various materials for pollen wall formation on the surface of pollen grains. So far, by using in situ hybridization [9]–[15] or microarrays [16], [17], many genes expressed in anthers have been identified in *Arabidopsis* [9], [13], [17], *Brassica napus* [10], rice [11], [12], [14], and *Lotus japonicus* [15], [16]. These studies have revealed the complex patterns of gene expression in both the gametophytic pollen/microspore and sporophytic tapetum cells, which are different but influence each other; consequently, it has been difficult to analyze the precise regulatory interactions of gene expression between pollen and tapetum by examination of the whole anther transcriptome In this context, transcriptome data from separated pollen and tapetum cells are necessary to compare with the large sets of other microarray data in order to achieve co-expression network analysis of male gametophyte development in plants. Recently, we have performed transcriptome analysis using 44 K microarrays with RNAs extracted independently from pollen and tapetum cells within the rice anther by laser microdissection (LM) technology [18], [19].

By using our LM-microarray [18], [19] and other publicly available microarray datasets [7], in this study, we have conducted comprehensive co-expression analysis and constructed the co-expression subnetworks responsible for two important biological events during anther development, meiosis and pollen wall synthesis. The meiosis network contained many putative meiotic genes as well as known meiotic genes, which could play meiotic roles such as meiosis initiation and recombination. Furthermore, from the pollen wall synthesis network, we efficiently identified several candidate genes involved in the sporopollenin biosynthesis pathway. Taken together, our co-expression network has the potential to be a powerful resource to dissect various important biological events and/or to isolate novel genetic factors regulating development and differentiation of the male reproductive organ in plants at a system biology level. In addition, this study is an important development as it is one of the few demonstrations of the efficiency of co-expression network analysis in rice.

## Results

### Dataset content

We have previously conducted 44 K agilent LM-microarray analysis of the microspore/pollen and tapetum cells of *japonica* rice, *Oryza sativa* cv. Nipponbare, during anther development from meiosis to tricellular pollen stages [18], [19]. In addition to the above study, a LM sample in premeiosis stage was prepared in this study, for more precise gene expression profiling during anther development. The overall LM-microarray data, including three novel microarray datasets, has been deposited at the Gene Expression Omnibus (GEO; http://www.ncbi.nlm.nih.gov/geo/; accession number GSE 29217). A detailed procedure for the LM-microarray is described in our previous reports [18], [19].

Recently, co-expression analysis datasets have become a powerful tool to extract the transcriptional networks of genes involved in various biological events in plants [20]. Therefore, we conducted microarray-based co-expression analysis using our LM-microarray data to gain biological insights into gene expression networks mediating rice anther development. Because popular methods that derive regulatory networks from gene expression data, in general, require large amounts of gene expression data, here we used 143 spatiotemporal expression profiles of 48 various vegetative, and reproductive organs and tissues of rice deposited in RicexPro [7], in addition to our 33 LM-microarray data of rice anthers [18], [19] (Table S1).

**Figure 1:**
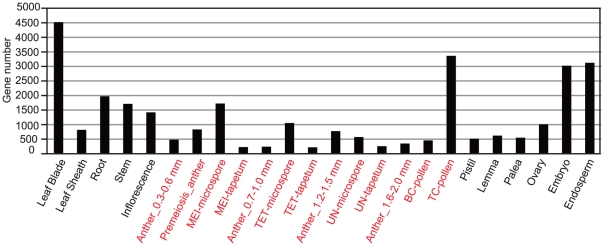
Distribution of rice genes with tissue-preferential expression in the 176 microarray datasets. The 29,864 distinct rice genes on a 44K microarray were classified into 24 spatiotemporal categories (different stages and tissues including LM-separated anther tissues) according to the highest signal intensity among the 176 microarray datasets. Gene number indicates the number of genes in each category. The spatiotemporal categories restricted to the anther are indicated in red. MEI, meiosis; TET, tetrad; UN, uninuclear; BC, bicellular; TC, tricellular.

A 44K agilent microarray slide contains 29,864 distinct rice genes. Using the 176 microarray, first, we examined the spatiotemporal expression pattern by identifying the highest signal intensity for each gene from different stages and organs (Figure 1, Tables S2, S3, S4, S5, S6, S7, S8, S9, S10, S11, S12, S13, S14, S15, S16, S17, S18, S19, S20, S21, S22, S23, S24, S25). Among the 29,864 genes analyzed, about 10,600 genes were found to be expressed preferentially in at least one of the anther developmental stages or tissues (Figure 1, Tables S7, S8, S9, S10, S11, S12, S13, S14, S15, S16, S17, S18, and S19 for array data of genes preferentially expressed in anther). The remaining approximately 19,300 genes were expressed in the other 44 vegetative or reproductive tissues (Tables S2, S3, S4, S5, S6, S20, S21, S22, S23, S24, S25). Such a large proportion of anther preferentially expressed genes indicates that many genes are involved in anther development and, consequently, analysis of co-expression networks of anther preferentially expressed genes could potentially provide good examples to demonstrate the application of microarray datasets.

### Biological significance of expression similarity

**Figure 2:**
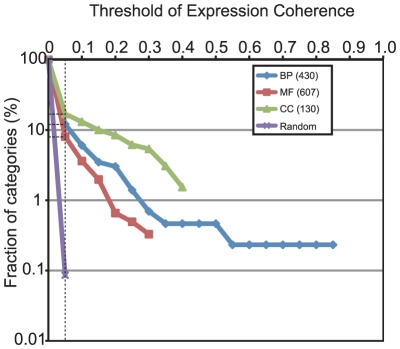
EC analysis of the 176 microarray data of rice. BP, MF, and CC represent Biological Process, Molecular Function, and Cellular Component groups of GO, respectively. The total number of categories in each GO group is indicated in parentheses. In the three GO groups (BP, blue; MF, red; and CC, green), the EC score was calculated for each GO category and compared with that of random sampling (purple) to estimate the statistical significant level. Fraction of categories (y axis) indicates the ratio of the number of GO categories with higher EC score than each threshold EC score (x axis) to the total number of GO categories.

We first conducted an expression coherence (EC) analysis using gene ontology (GO) terms to examine the biological significance of expression similarity in the 176 microarray datasets used in this study. EC analysis is a statistical analysis to determine whether gene members belonging to a predefined functional gene set are correlated with each other at the transcriptional level [21]. The EC score is a measure of the expression similarity within a set of predefined functional genes [21]; consequently, a higher EC score is obtained, when most of genes with the same GO term are co-expressed with each other. In our EC analysis, the EC scores of all three GO groups (Biological Process, BP; Molecular Function, MF; and Cellular Component, CC) were higher than that expected by random sampling (Figure 2). Among the three GO groups, CC showed the highest expression similarity, in which approximately 17% of categories exhibited higher EC scores than random sampling at threshold EC score of 0.05, while approximately 12% and 8% did so in BP and MF, respectively (dotted line in Figure 2). For genes annotated according to the BP group, the “photosynthesis, light harvesting” category showed the highest EC scores (EC = 0.868, 17 genes), whereas the scores of the categories “small ribosomal subunit” (EC = 0.433, 21 genes) and “threonine-type endopeptidase activity” (EC = 0.322, 24 genes) were highest in the CC and MF groups (Table S26). These results confirm that the levels of expression similarity estimated from the microarray datasets in this study are good reflections of functional similarity.

### Construction of the meiosis-specific network

By using the Pearson correlation coefficient (PCC) method with the mutual rank (MR)-based cut-off (see Materials and Methods), we identified the final dataset of rice co-expression networks generated from all the 176 microarray data used in this study, which contains 24,259 genes (nodes) and 60,449 genes pairs (edges). From this entire dataset of the rice co-expression network, we are able to construct a subnetwork around gene(s) of interest input as guide(s).

**Figure 3:**
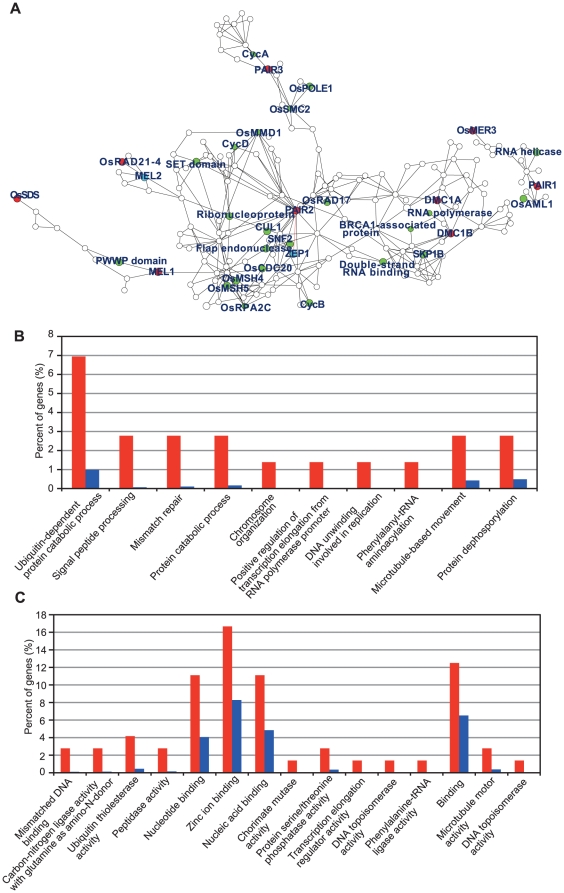
The meiosis-specific co-expression network in rice. (A) The co-expression subnetwork constructed using 9 reported rice meiotic genes as guide genes (red circles). The network contains the most-recently reported meiotic genes (blue circles), putative meiotic genes (green circles) and other genes (open circles). A red bold line indicates the direct connection of *PAIR2* and *ZEP1* in the co-expression network. A link between two nodes indicates direct interaction with PCC >0.64 and MR <10. The subnetwork vicinity is extracted by taking 2 steps out from a guide gene. (B, C) Percentage of genes classified in enriched GO terms of BP (B) or MF (C) for the genes in the meiosis-specific network (red) relative to for 11,456 genes (blue) that had at least one connection at the PCC (>0.64) and MR (<10) thresholds. Enrichment analysis is performed using the hypergeometric distribution (P value <0.05).

To examine whether our co-expression network analysis can identify useful transcriptional networks related to anther development, we first constructed a co-expression subnetwork centered on 9 known meiotic genes (guide genes; red circles in Figure 3A), *PAIR1* [22], *PAIR2* [23], *PAIR3* [24], *MEL1* [25], *OsRAD21*–*4* [26], *DMC1A* and *1B* [27], *OsMER3/OsRCK* [28], and *OsSDS* [28], whose functions in meiosis have been experimentally demonstrated in rice. The expression profiles of these nine genes after normalized by the VSN algorism (Figure S1A) are well corresponding to the previous results of their expression patterns: indicating that the VSN normalization was a suitable method for the construction of co-expression network. The meiosis-specific network consisted of a large main cluster with the 7 guide genes, and two independent minor clusters of *OsMER3* and *PAIR1* (Figure 3A), which in total contained 187 genes and 346 gene interactions (Table S27). The main cluster also contained *ZEP1* (rice *ZYP1* ortholog) and *MEL2* (blue circles in Figure 3A), which have most recently been reported as rice meiotic genes [29], [30]. Moreover, *PAIR2* and *ZEP1* [23], [29], whose products are both constituents of the synaptonemal complex, were directly connected in the network (red bold lines in Figure 3A). Good association of the 7 meiotic guide genes with each other and with the additional 2 meiotic genes in the main expression cluster indicates that genes involved in meiosis could be strictly co-regulated at the expression level, and thus the meiotic events are potentially good subjects for co-expression analysis. This is also supported by the results of GO analysis (Figures 3B and 3C, Table S28), in which DNA-repair-related GO terms (such as “chromosome organization,” “DNA unwinding involved in replication,” “Mismatch repair,” and “Mismatched DNA binding”) appear frequently in genes in the meiosis-specific network.

Known or putative meiotic genes in the subnetwork are listed in Table 1. The meiosis-specific network contained *cyclin* genes and other potential rice orthologs of meiotic genes (Figure 3A). In the network, genes related to meiotic recombination (e.g. *DMC1s* and *OsMSH4/5*) are located near a core of the network, and genes functioning in other meiotic events (e.g. *MEL1*, *PAIR3*, *OsRAD21*–*4*, and *OsSDS*) surround them with the small independent cluster of *PAIR1* (Figure 3A). The independent small cluster of *OsMER3* (a meiotic recombination-related gene) is due to its higher vegetative expression, which could indicate a bifunctional role in meiosis and other biological events.

**Table 1:** Known or putative meiotic genes in the meiosis-specific subnetwork.

RAP-ID	Gene_name	Description	Category
**Transition from mitosis into meiosis**			
Os03g0800200	MEL1	PAZ domain containing protein	Guide gene
Os12g0572800	MEL2	RNA recognition motif family protein	Known meiotic gene
Os01g0907900	OsAML1	AML1, putative, expressed	Known meiotic gene
Os05g0129900	RNA polymerase	RNA polymerase II-associated protein 3	Putative
Os08g0399500	SET domain	YDG/SRA domain containing protein	Putative
Os05g0122500	PWWP domain	PWWP domain containing protein	Putative
Os08g0384100	Double-stranded RNA binding	double-stranded RNA binding motif containing protein	Putative
Os02g0730100	Ribonucleoprotein	pre-mRNA processing ribonucleoprotein, binding region	Putative
**Homologus pairing**			
Os03g0106300	PAIR1	PAIR1	Guide gene
**Synapsis**			
Os09g0506800	PAIR2	retrotransposon protein, putative, SINE subclass	Guide gene
Os04g0452500	ZEP1	synaptonemal complex protein 2	Known meiotic gene
Os10g0405500	PAIR3	expressed protein	Guide gene
**Meiotic replication and chrmosome structure control**			
Os02g0511900	OsPOLE1	POLE1 - Putative DNA polymerase epsilon catalytic subunit	Putative
Os09g0521900	Flap endonuclease	flap endonuclease	Putative
Os05g0580500	OsRAD21-4	Rad21 / Rec8 like protein, putative	Guide gene
Os01g0904400	OsSMC2	chromosome segregation protein	Putative
Os06g0693300	OsRPA2C	RPA2C	Putative
**Meiotic recombination**			
Os12g0143800	DMC1A	DNA repair protein Rad51	Guide gene
Os11g0146800	DMC1B	DNA repair protein Rad51	Guide gene
Os04g0648500	BRCA1-associated protein	BRCA1-associated protein	Putative
Os03g0242100	OsRAD17	cell cycle checkpoint protein RAD17	Putative
Os05g0389800	RNA helicase	ATP-dependent RNA helicase	Putative
Os07g0636200	SNF2	SNF2 family N-terminal domain containing protein	Putative
Os02g0617500	OsMER3	DEAD/DEAH box helicase domain containing protein	Guide gene
Os07g0486000	OsMSH4	mutS family domain IV containing protein	Known meiotic gene
Os05g0498300	OsMSH5	mutS domain V family protein	Known meiotic gene
**Meiotic progression**			
Os03g0225200	OsSDS	cyclin	Guide gene
Os01g0233500	CycA (Cyclin A)	cyclin-A1	Putative
Os01g0281200	CycB (Cyclin B)	cyclin	Putative
Os07g0556000	CycC (Cyclin D)	cyclin	Putative
Os01g0935300	CUL1	cullin-1	Putative
Os07g0624900	SKP1B	SKP1-like protein 1B	Putative
Os03g0716200	OsMMD1	PHD-finger domain containing protein	Known meiotic gene
Os09g0242300	OsCDC20	WD domain, G-beta repeat domain containing protein	Putative

Among genes related to “transition from mitosis into meiosis” in Table 1, *MEL1* and *MEL2* encode ARGONAUTE and RNA-recognition-motif protein, respectively [24], [30], and putative *OsAML1* (Os01g0907900) also encodes an RNA-binding protein [31], which is highly similar to *MEI2* of *Schizosaccharomyces pombe* [32]. Together with PWWP- and SET-domain proteins (Table 1), *MEL1*, *MEL2* and *OsAML1* might regulate the initiation of meiosis in rice through molecular mechanisms related to RNA metabolism, chromatin remodeling and/or transcriptional regulation. On the other hand, genes related to “meiotic recombination” are well integrated in the network (Figure 3A, Table 1). Co-expression of recombination-related genes in the network is consistent with previous reports that *DMC1A* and *DMC1B* co-ordinate with *RAD17* [33], and that *MSH4* and *MSH5* function as a complex [34].

In addition, it can be concluded from the GO analysis (Figure 3B and 3C, Table S28) that various additional meiotic genes could be included in the network as unknown genes (open circles in Figure 3A). This indicates that novel meiotic genes could be mined from the present data, and that novel interactions between known meiotic genes could also be identified from the unknown-gene-mediated connections in the network. Meiosis is an extremely specialized biological event, thus co-expression network analysis has the potential to achieve maximum success in the identification of gene interactions.

### Construction of the pollen wall synthesis network

**Figure 4:**
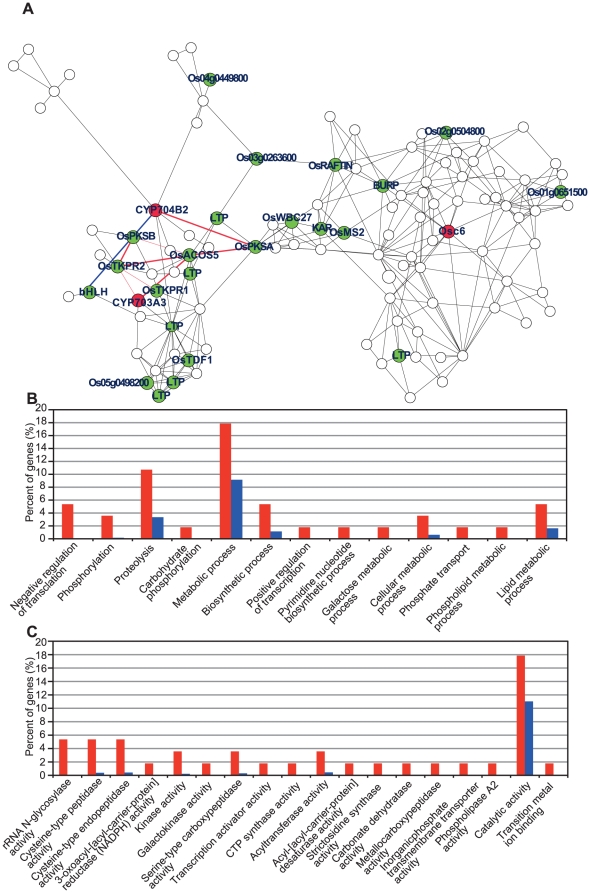
The pollen wall synthesis co-expression network in rice. (A) The co-expression subnetwork constructed using 3 reported rice genes for sporopollenin biosynthesis or transport as guide genes (red circles). The network contains putative genes related to pollen wall synthesis (green circles). Red bold lines indicate the direct connection of *CYP703A3*, *CYP704B2*, *OsACOS5*, *OsPKS*s, and *OsTKPR*s in the co-expression network. A blue bold line indicates the direct connection between Os01g0293100 (a bHLH-type transcription factor) and *CYP704B2*. A link between two nodes indicates direct interaction with PCC >0.64 and MR <10. The subnetwork vicinity is extracted by taking 2 steps out from a guide gene. (B, C) Percentage of genes classified in enriched GO terms of BP (B) or MF (C) for the genes in the meiosis-specific network (red) relative to for 11,456 genes (blue) that had at least one connection at the PCC (>0.64) and MR (<10) thresholds. Enrichment analysis is performed using the hypergeometric distribution (P value <0.05).

As a second example system, we analyzed the pollen wall synthesis network to systemically identify the genes that modulate the formation of the outer pollen wall, the exine. Previous biochemical analyses have shown that the exine layer consists mainly of sporopollenin, a polymer of phenylpropanoid and lipidic monomers covalently linked by ether and ester linkages [35]–[37]. When three genes for rice sporopollenin biosynthesis or transport (*CYP703A3*, *CYP704B2*, and *Osc6*) were selected as guide genes [38]–[40], a major subnetwork covering all guide genes was identified, which consisted of 108 genes and 278 gene interactions (Figure 4A, Table S29). Again, the expression profiles of these three genes after normalized by the VSN algorism (Figure S1B) are well corresponding to the previous results of their expression patterns. Classification according to GO terms showed that this subnetwork was significantly enriched with fatty acid and several metabolic related terms, including “phospholipid metabolic process”, “lipid metabolic process”, “3-oxoacyl-[acyl-carrier-protein] reductase (NADPH) activity”, and “phospholipase A2 activity” (Figure 4B and 4C, Table S30). This is clearly consistent with previous knowledge of sporopollenin biochemistry [35]–[37], indicating that this co-expression network reflects the underlying molecular mechanism for exine formation.

**Figure 5:**
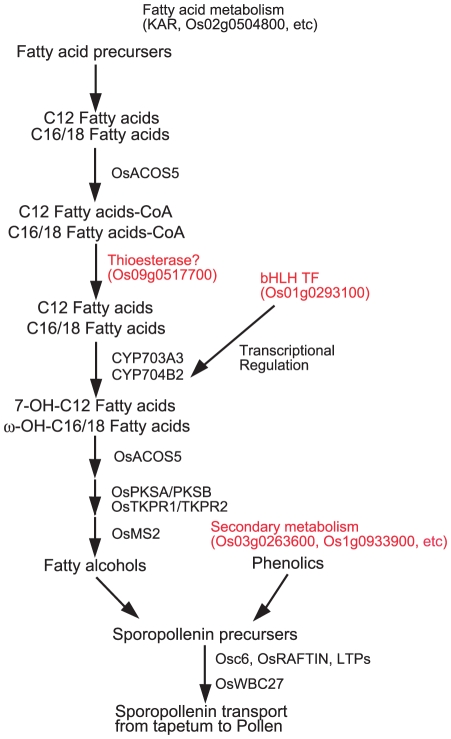
The current model of the sporopollenin biosynthetic pathway.

In addition to the above three guide genes, this network contained several fatty acid metabolic genes, such as *OsMS2*, *OsACOS5* (*ACYL-COA SYNTHETASE5*), *OsPKS*s (*POLYKETIDE SYNTHASE*), and *OsTKPRs* (*TETRAKETIDE a-PYRONE REDUCTASE*), homologs of which have been demonstrated to be important for sporopollenin biosynthesis in *Arabidopsis* [41]–[44] (Figure 4A; Table 2). In the latest model of the sporopollenin biosynthetic pathway [44], [45], fatty acid precursors are esterified to CoA by ACOS5, and then hydroxylated by CYP703A and CYP704B to produce substrates of the subsequent ACOS5 reaction (Figure 5). Subsequently, ACOS5, PKSs, TKPRs, and MS2 mediate the biochemical reactions to produce sporopollenin precursors via fatty alcohols (Figure 5). Consistently, our pollen wall specific network confirmed significant gene interactions between these molecular players (red bold lines in Figure 4A). Furthermore, in order to identify an as-yet-unknown specific thioesterase producing the CYP703A/CYP704B substrates, we compared the expression patterns of 15 rice thioesterase genes with those of the sporopollenin biosynthetic genes, and detected one thioestrase gene (Os09g0517700) that was co-expressed with *OsPKSB* and *OsTKPR2* at the significant PCC level (Table 3). Although Os09g0517700 was not found on the pollen wall synthesis subnetwork (Figure 4A) because of higher MR values, it might play an important role in the sporopollenin biosynthetic pathway.

**Table 2:** Known or putative rice genes involved in pollen wall synthesis in the pollen wall synthesis subnetwork.

RAP-ID	Gene_name	Description	Category
The synthesis of Fatty acids precursers			
Os12g0242700	KAR	3-oxoacyl-reductase, chloroplast precursor	Putative
Os02g0504800	Os02g0504800	PE-PGRS family protein	Putative

Sporopollenin biosynthesis			
Os04g0310800	OsACOS5	AMP-binding domain containing protein	Known pollen wall synthesis gene
Os08g0131100	CYP703A3	cytochrome P450	Guide gene
Os03g0168600	CYP704B2	cytochrome P450	Guide gene
Os10g0484800	OsPKSA	stilbene synthase	Known pollen wall synthesis gene
Os07g0411300	OsPKSB	chalcone and stilbene synthases	Known pollen wall synthesis gene
Os08g0515900	OsTKPR1	dihydroflavonol-4-reductase	Known pollen wall synthesis gene
Os01g0127500	OsTKPR2	dihydroflavonol-4-reductase	Known pollen wall synthesis gene
Os03g0167600	OsMS2	male sterility protein	Known pollen wall synthesis gene
Os090517700	Os090517700	acyl-coenzyme A thioesterase 10, mitochondrial precursor	Putative

The transcriptional regulation of sporopollenin biosynthetic genes			
Os01g0293100	bHLH	helix-loop-helix DNA-binding domain containing protein	Putative
Os03g0296000	OsTDF1	MYB family transcription factor	Known pollen wall synthesis gene

The secondary metabolism			
Os01g0933900	Os01g0933900	glutathione S-transferase	Putative
Os01g0651500	Os01g0651500	wax synthase isoform 3	Putative
Os03g0263600	Os03g0263600	strictosidine synthase	Putative
Os05g0498200	Os05g0498200	glycine-rich cell wall protein	Putative
Os04g0449800	Os04g0449800	alpha/beta hydrolase fold	Putative

The transport of Sporopollenin			
Os11g0582500	Osc6	LTPL68 - Protease inhibitor/seed storage/LTP family protein precursor	Guide gene
Os07g0244900	LTP	LTPL54 - Protease inhibitor/seed storage/LTP family protein precursor	Putative
Os09g0525500	LTP	LTPL45 - Protease inhibitor/seed storage/LTP family protein precursor	Putative
Os03g0357500	LTP	LTPL1 - Protease inhibitor/seed storage/LTP family protein precursor	Putative
Os08g0546300	LTP	LTPL44 - Protease inhibitor/seed storage/LTP family protein precursor	Putative
Os08g0545800	LTP	LTPL97 - Protease inhibitor/seed storage/LTP family protein precursor	Putative
Os01g0691300	LTP	LTPL150 - Protease inhibitor/seed storage/LTP family protein precursor	Putative
Os08g0496800	OsRAFTIN	BURP domain containing protein	Known pollen wall synthesis gene
Os09g0480900	BURP	BURP domain containing protein	Putative
Os06g0607700	OsWBC27	ABC-2 type transporter domain containing protein	Known pollen wall synthesis gene

**Table 3:** Expression similarities between 15 putative thioesterase genes and 7 sporopollenin biosynthetic genes in rice.

RAP-ID	OsTKPR2	CYP704B2	OsACOS5	OsPKSB	CYP703A3	OsTKPR1	OsPKSA
Os01g0229500	0.025	−0.136	0.090	−0.067	−0.090	−0.038	0.076
Os01g0229600	−0.359	−0.558	−0.397	−0.449	−0.486	−0.399	−0.406
Os01g0882000	−0.098	0.174	−0.005	−0.076	−0.027	−0.019	−0.018
Os01g0882100	−0.041	0.156	−0.013	0.008	0.107	−0.069	0.041
Os02g0521700	0.041	−0.085	0.043	0.072	0.064	0.030	0.027
Os03g0691400	−0.462	−0.414	−0.480	−0.481	−0.512	−0.398	−0.478
Os04g0436100	−0.230	−0.380	−0.274	−0.311	−0.394	−0.197	−0.264
Os04g0553300	0.375	0.601	0.403	0.439	0.533	0.336	0.431
Os04g0558400	−0.252	−0.209	−0.293	−0.304	−0.214	−0.278	−0.234
Os05g0137700	0.315	0.039	0.286	0.282	0.118	0.319	0.110
Os07g0462700	−0.087	−0.395	−0.119	−0.170	−0.330	−0.060	−0.191
Os07g0463500	0.010	0.005	−0.048	−0.093	0.081	0.001	0.102
Os09g0517700	0.672*	0.242	0.584	0.664*	0.541	0.598	0.460
Os02g0660800	N.T.	N.T.	N.T.	N.T.	N.T.	N.T.	N.T.
Os04g0553100	N.T.	N.T.	N.T.	N.T.	N.T.	N.T.	N.T.

The PCC value of each gene pair was used as a measure of expression similarity.

Values with asterisk indicate a higher PCC value than the threshold value, 0.64.

N.T., Not tested because of no probe in microarray slide.

In addition to the previously characterized genes, several novel genes, whose products participate in fatty acid metabolism, secondary metabolism, and lipid transport, were identified within this network (Figure 4A and Table 2). It is probable that these genes are associated with novel metabolic processes related to sporopollenin biosynthesis or sporopollenin transfer from tapetal cells to anther locules (Figure 5).

It has also been reported that some MYB and bHLH transcriptional factors, including GAMYB, AMS, UDT1, and AtMYB103, regulate the expression of sporopollenin biosynthetic genes [38], [46]–[48]. The 108 co-expressed genes in the pollen wall synthesis network included the two candidates (Os01g0293100 and Os03g0296000) for the transcriptional regulation for sporopollenin biosynthesis (Figure 4A and Table 2). Os03g0296000 is a rice homolog of *Arabidopsis MYB35* (*TDF1*) [49] responsible for tapetum development, whereas Os01g0293100 is a novel bHLH type transcription factor, not previously studied. In this network, Os01g0293100 is directly connected to *CYP704B2* (blue line in Figure 4A), suggesting that Os01g0293100 could be involved in sporopollenin biosynthesis via modulating *CYP704B2* expression (Figure 5). Thus, the pollen wall synthesis network in the present co-expression analysis efficiently provides us with useful information to identify novel gene functions and interactions.

## Discussion

### Co-expression network analysis in plants

In *Arabidopsis*, useful databases for co-expression analysis are being constructed, and several novel genes have been identified from these data. Aoki et al. [3] and Obayashi et al. [50] listed the available co-expression databases in *Arabidopsis*, including CressExpress [51], CoP [52], ACT [53], GeneCAT [54], and ATTED-II [55]. As described in these review articles, novel genes for enzymes involved in metabolic pathways, signal factors of biological events (including transcription factors), and components of protein complexes have been identified by using the publicly available databases or analyzing a laboratory's own co-expression networks. For example, *irregular xylem8* (*IRX8*) and *irregular xylem13* (*IRX13*), which are involved in secondary cell wall formation, were identified from co-expression analysis guided by members of a cellulose synthase (*CESA*) family [4]. *IRX8* encodes glycosyltransferase (GT8), which is involved in pectin synthesis, whereas *IRX13* encodes an unknown protein similar to an arabinogalactan protein. Involvement of *IRX8* and *IRX13* in secondary cell wall formation was demonstrated by T-DNA insertion mutagenesis. Similarly, Oikawa and co-workers [56] conducted a comparative co-expression analysis between *Arabidopsis* and rice, and proposed a model for secondary cell wall formation of *Arabidopsis* and rice, based on the combined approach of co-expression and predicted cellular localization. More recently, by using *Arabidopsis* co-expression networks, *UGP3*, a gene coding for UDP-glucose pyrophosphorylase 3 implicated in sulfolipid biosynthesis [57], *NAI2* implicated in endoplasmic reticulum body formation [58], *BTS*, a *BRUTUS* gene coding for bHLH-type transcription factor co-expressed with *POPEYE* in iron homeostasis [59], and *STOMAGEN*, encoding secretory peptide regulating stomatal density [60], were successfully identified. Thus, co-expression network analysis is a powerful tool for the identification of novel *Arabidopsis* genes, and such an approach is undoubtedly also applicable to rice research.

In the present study, we performed co-expression network analysis of rice genes by using 176 datasets of 44 K agilent microarrays (including 33 LM-microarray datasets of the male reproductive organs), and constructed two different co-expression subnetworks (meiosis-specific and pollen-wall-synthesis networks) to demonstrate the efficiency of this technique. Integrative application of such comprehensive microarray data enabled us to construct high-resolution networks (Figures 3A and 4A), which included useful information for the dissection of gene regulation in biological processes. This demonstrates that we can now perform co-expression screening to pursue our interests in specific genes or biological events in rice, as well as in *Arabidopsis*, by the use of our reliable network datasets and the other effective rice databases reported to date [61]–[64].

To the best of our knowledge, this is one of the few reports of the application of co-expression network analysis to specific biological processes in rice. Four rice co-expression databases are now publicly available. In the most recent version of ATTED-II, the co-expression network can be constructed by using the mutual rank of the Pearson's correlation coefficient (PCC) in rice as well as in *Arabidopsis* [61]. Ficklin et al. [62] demonstrated application of the Gene Co-expression Network Browser, a website providing a rice co-expression network constructed by random matrix theory and weighted correlation network analysis. In addition, RiceArrayNet [63], based on PCC, and OryzaExpress [64], based on correspondence analysis, are available as rice co-expression databases. These four recent papers [61]–[64] reported the availability of the rice co-expression databases, but did not provide a demonstration of the identification of genes in relation to specific biological events. Therefore, our present report is a valuable addition to the understanding of the utility of rice co-expression network analysis, together with discussion of specific examples of successful construction of meiosis-specific and pollen-wall-synthesis networks.

### Application of co-expression network analysis in sexual reproduction research

Our co-expression analysis included the LM microarray data of male reproductive organs, which can discriminate transcriptomes of gametophytic pollen/microspore cells and sporophytic tapetum cells in the anther, and will provide more precise information on transcriptional regulation of anther genes than has been available previously. None of the examples of gene identification using *Arabidopsis* co-expression networks described above focus on the microarray databases of reproductive organs, conditions or developmental stages [4], [56]–[60]. Hence, our co-expression network datasets, focusing on male gametophyte development, shed light on a new and interesting aspect of the potential of co-expression analysis. It may be interesting to apply co-expression network analysis to other LM microarray datasets targeted at specific tissues and cells.

Our meiosis-specific and pollen-wall-synthesis networks reveal that many previously unidentified genes are co-expressed in these specific reproductive events. A reverse genetics approach to the study of these genes will lead to identification of novel meiosis-specific and pollen-wall-synthesis genes. Because there are many interesting and specific biological events during male gametophyte development, the very large co-expression datasets presented here will provide an excellent opportunity for plant researchers to study the molecular biology of sexual reproduction in rice.

## Materials and Methods

### Plant materials

Rice (*O. sativa* L. ssp. *japonica* cv. Nipponbare) plants were grown in a greenhouse under normal conditions. Anthers at the premeiosis stage were collected after their developmental stages were confirmed by DAPI staining using one of the six anthers of each flower.

### LM

Anthers were fixed in Farmer's fixative (ethanol: acetate = 3∶1) overnight at 4°C. Dehydration and paraffin embedding were performed using a microwave processor [31]. Paraffin-embedded sections were cut to a thickness of 16 µm and mounted on PEN membrane glass slides (Molecular Devices, Sunnyvale, CA, USA) for LM. To remove paraffin, slides were immersed in 100% xylene (twice), 50% xylene/50% ethanol, and 100% ethanol (v/v), for 5 min at each step and then air-dried completely at room temperature. Three or four individual flowers were used for each LM experiment. LM was performed using the Veritas Laser Microdissection System LCC1704 (Molecular Devices). Selected areas were captured by an infrared (IR) laser onto CapSure Macro LCM Caps (Molecular Devices) and subsequently cut by a UV laser. The target cells that fused to the LCM cap were collected by removing the cap from the tissue section.

### Microarray analysis

Total RNAs were extracted from the LM cells with a PicoPure™ RNA isolation kit (Molecular Devices), quantified with a Quant-iT™ RiboGreen RNA reagent and kit (Invitrogen, Carlsbad, CA, USA), and subjected to the rice 44 K oligo microarray (Agilent Technologies, Santa Clara, CA, USA), which contains ∼42,000 oligonucleotides based on the nucleotide sequence and full-length cDNA of the Rice Annotation Project Database (RAP-DB) [65]. Fluorescent probe labeling, hybridization, and scanning were performed according to the manufacturer's instructions (Agilent Technologies), with slight modifications. Each experiment was performed three times using independently isolated samples (three biological replicates). Feature extraction software (Agilent Technologies) was used to delineate and measure the signal intensity of each spot in the array. All microarray data, including our microarray data and publically available data, were statistically normalized by variance stabilization normalization (VSN) using R software (http://www.r-project.org/). The normalized signal intensities were then transformed to log base 2. All microarray data is MIAME compliant and the raw data from this study have been deposited in Gene Expression Omnibus (GEO; http://www.ncbi.nlm.nih.gov/geo/) under accession number GSE 29217.

### GO and EC analyses

GO terms were obtained for rice genes to classify their biological functions [66]. GO terms for GO enrichment analysis and EC analysis were retrieved from the RAP-DB. EC analysis was performed as previously described [21], [67], with the following modifications: genes with the same GO term were used as a set of predefined functional genes (each set contained 10 or more genes). EC reports the fraction of gene pairs per GO category that show elevated co-expression. Here, the PCC was utilized as a measure for expression similarity, and we used a PCC threshold of 0.64, corresponding to the 99^th^ percentile of random PCC distribution derived for 1,000 random genes (approximately 1,000*999*0.5 gene pairs). To calculate the random EC for GO categories, random gene sets were sampled with the same size as the category under investigation. For GO enrichment analysis, the statistical significance of GO enrichment within co-expressed genes was evaluated against a background set consisting of 11,456 genes with at least one connection from the dataset of rice co-expression networks using the hypergeometric distribution without a multiple-testing correlation, and P values <0.05 were set as the significant threshold.

### Co-expression analysis

To construct co-expression networks, we calculated the PCC values for all combinations of unique 29,864 probes present on the rice 44 K oligo microarray. We estimated two PCC thresholds of 0.48 and 0.65, corresponding to the 95^th^ and 99^th^ percentile of random PCC distribution derived for 1,000 random genes (approximately 1,000*999*0.5 gene pairs). In some previous co-expression studies [3], [68], any two genes with a PCC value greater than 0.6 between their expression profiles were considered as co-expressed genes. Therefore, we set a PCC threshold of 0.64 corresponding to the 99^th^ percentile of random PCC distribution, as described above. For all gene pairs with a significant PCC value, we also calculated MR values between them as another value of co-expression measure to further reduce the number of false positives, according to a previous report [69]. Finally, if the absolute value of MR was lower than 10, the gene pair was considered as a significant connection for the co-expression network. These calculations were conducted using the R/Bioconductor. In extracting meiosis or pollen-wall subnetwork datasets, the network vicinity was extracted by taking 2 steps out from a guide gene, as described previously by Mutwil et al. [70]. The network was illustrated using the program Cytoscape.

## Supporting Information

Figure S1 **Representative tissue expression profile of the guide genes after normalization by VSN algorism.**Nine meiosis guide genes (A) are preferentially expressed in anther and microspore at meiosis (MEI) stage, whereas three pollen wall guide genes (B) are expressed in microspore and tapetum at tetrad (TET) or uninuclear (UNI) stage. The bar at top represents the average signal intensity (log2). Details on the individual samples can be found in Table S1. All 176 microarray data used for co-expression analysis show a similar expression pattern to those of these guide genes, respectively (Tables S2, S3, S4, S5, S6, S7, S8, S9, S10, S11, S12, S13, S14, S15, S16, S17, S18, S19, S20, S21, S22, S23, S24, S25).
(EPS)

Table S1 **GEO IDs of the 176 microarray profiles in this study.**
(XLS)

Table S2 **Microarray data of Leaf Blade preferentially expressed genes.**
(RAR)

Table S3 **Microarray data of Leaf Sheath preferentially expressed genes.**
(XLS)

Table S4 **Microarray data of Root preferentially expressed genes.**
(XLS)

Table S5 **Microarray data of Stem preferentially expressed genes.**
(XLS)

Table S6 **Microarray data of Inflorescence preferentially expressed genes.**
(XLS)

Table S7 **Microarray data of Anther_0.3–0.6** **mm preferentially expressed genes.**
(XLS)

Table S8 **Microarray data of Premeiosis anther preferentially expressed genes.**
(XLS)

Table S9 **Microarray data of Meiosis (MEI)-microspore preferentially expressed genes.**
(XLS)

Table S10 **Microarray data of Meiosis (MEI)-tapetum preferentially expressed genes.**
(XLS)

Table S11 **Microarray data of Anther_0.7–1.0** **mm preferentially expressed genes.**
(XLS)

Table S12 **Microarray data of Tetrad (TET)-microspore preferentially expressed genes.**
(XLS)

Table S13 **Microarray data of Tetrad (TET)-tapetum preferentially expressed genes.**
(XLS)

Table S14 **Microarray data of Anther_1.2–1.5** **mm preferentially expressed genes.**
(XLS)

Table S15 **Microarray data of Uninuclear (UN)-microspore preferentially expressed genes.**
(XLS)

Table S16 **Microarray data of Uninuclear (UN)-tapetum preferentially expressed genes.**
(XLS)

Table S17 **Microarray data of Anther_1.6–2.0** **mm preferentially expressed genes.**
(XLS)

Table S18 **Microarray data of Bicellular (BC)-pollen preferentially expressed genes.**
(XLS)

Table S19 **Microarray data of Tricellular (TC)-pollen preferentially expressed genes.**
(RAR)

Table S20 **Microarray data of Pistil preferentially expressed genes.**
(XLS)

Table S21 **Microarray data of Lemma preferentially expressed genes.**
(XLS)

Table S22 **Microarray data of Palea preferentially expressed genes.**
(XLS)

Table S23 **Microarray data of Ovary preferentially expressed genes.**
(XLS)

Table S24 **Microarray data of Embryo preferentially expressed genes.**
(RAR)

Table S25 **Microarray data of Endosperm preferentially expressed genes.**
(RAR)

Table S26 **EC scores for different GO categories.**
(XLS)

Table S27 **PCC and MR values of all gene pairs in the meiotic subnetwork.**
(XLS)

Table S28 **Frequency of GO categories for genes in the meiotic subnetwork.**
(XLS)

Table S29 **PCC and MR values of all gene pairs in the pollen wall synthesis subnetwork.**
(XLS)

Table S30 **Frequency of GO categories for genes in the pollen wall synthesis subnetwork.**
(XLS)
